# Identification and characterization of the zinc-regulated transporters, iron-regulated transporter-like protein (ZIP) gene family in maize

**DOI:** 10.1186/1471-2229-13-114

**Published:** 2013-08-08

**Authors:** Suzhen Li, Xiaojin Zhou, Yaqun Huang, Liying Zhu, Shaojun Zhang, Yongfeng Zhao, Jinjie Guo, Jingtang Chen, Rumei Chen

**Affiliations:** 1 Department of Agronomy, Agricultural University of Hebei/Hebei Sub-center of Chinese National Maize Improvement Center, Baoding 071001, China; 2 Department of Crop Genomics & Genetic Improvement, Biotechnology Research Institute, Chinese Academy of Agricultural Sciences, Beijing 100081, China

**Keywords:** Embryo, Endosperm, Expression profiling, Zinc, Iron, Zinc-regulated transporters, Iron-regulated transporter-like protein (ZIP), Subcellular localization, Yeast complementation, Maize

## Abstract

**Background:**

Zinc (Zn) and iron (Fe) are essential micronutrients for plant growth and development, their deficiency or excess severely impaired physiological and biochemical reactions of plants. Therefore, a tightly controlled zinc and iron uptake and homeostasis network has been evolved in plants. The Zinc-regulated transporters, Iron-regulated transporter-like Proteins (ZIP) are capable of uptaking and transporting divalent metal ion and are suggested to play critical roles in balancing metal uptake and homeostasis, though a detailed analysis of *ZIP* gene family in maize is still lacking.

**Results:**

Nine ZIP-coding genes were identified in maize genome. It was revealed that the ZmZIP proteins share a conserved transmembrane domain and a variable region between TM-3 and TM-4. Transiently expression in onion epidermal cells revealed that all ZmZIP proteins were localized to the endoplasmic reticulum and plasma membrane. The yeast complementation analysis was performed to test the Zn or Fe transporter activity of ZmZIP proteins. Expression analysis showed that the *ZmIRT1* transcripts were dramatically induced in response to Zn- and Fe-deficiency, though the expression profiles of other *ZmZIP* changed variously. The expression patterns of *ZmZIP* genes were observed in different stages of embryo and endosperm development. The accumulations of *ZmIRT1* and *ZmZIP6* were increased in the late developmental stages of embryo, while *ZmZIP4* was up-regulated during the early development of embryo. In addition, the expression of *ZmZIP5* was dramatically induced associated with middle stage development of embryo and endosperm.

**Conclusions:**

These results suggest that *ZmZIP* genes encode functional Zn or Fe transporters that may be responsible for the uptake, translocation, detoxification and storage of divalent metal ion in plant cells. The various expression patterns of *ZmZIP* genes in embryo and endosperm indicates that they may be essential for ion translocation and storage during differential stages of embryo and endosperm development. The present study provides new insights into the evolutionary relationship and putative functional divergence of the *ZmZIP* gene family during the growth and development of maize.

## Background

Zinc and iron are essential for plant metabolism and development [1]. Zinc serves as a key structural motif in many proteins, including DNA-binding Zn-finger protein [2,3], RING finger proteins and LIM domain containing proteins [4], which play vital roles in controlling cellular processes such as growth, development and differentiation. An adequate Zn content enhances crop productivity [5]. Iron plays an important role in electron transfer in photosynthesis and respiration, though high concentration of intracellular iron may lead to elevated Fe^3+^/Fe^2+^ redox reactions and cause damage [6]. The deficiency of Zn and Fe decreases plant growth and affecting cereal production and grain quality [7], but excess Zn and Fe may cause significant toxicity to biological systems [8,9]. Therefore, plants have established a tightly controlled system to balance the uptake, utilization and storage of these metal ions [10,11]. Because Zn cannot diffuse across cell membrane, specific zinc transporters are required to transport Zn into cytoplasm [12,13]. In recent years, a number of metal transporters have been identified in plants, including the P_1B_-ATPase family, zinc-regulated transporter (ZRT), iron-regulated transporter (IRT)-like protein (ZIP), natural resistance-associated macrophage protein (NRAMP) family, and cation diffusion facilitator (CDF) family [14].

ZRT, IRT-like protein (ZIP) family has been characterized ubiquitously in organisms, including archaea, bacteria, fungi, plants and mammals, and has been demonstrated to be involved in metal uptake and transport [11]. ZIP proteins generally contribute to metal ion homeostasis by transporting cations into the cytoplasm [14]. Functional complementation in yeast indicated that ZIP proteins are able to transport various divalent cations, including Fe^2+^, Zn^2+^, Mn^2+^, and Cd^2+^[15]. The ZIP proteins consist of 309-476 amino acid residues with eight potential transmembrane domains and a similar membrane topology. There is also a variable region between TM-3 and TM-4, in which the amino- and carboxyl-terminals located on the outside surface of plasma membrane. The variable region contains a potential metal-binding domain rich in conserved histidine residues [15]. Several ZIP proteins have been identified in *Arabidopsis*[16]. AtIRT1 (Iron-regulate transporter 1) was the first member to be identified through functional complementation of a yeast mutant defective in iron uptake, and it encodes a major Fe transporter at the root surface in *Arabidopsis*[17-20]. Further analysis showed that the *irt1* mutant exhibited lethal chlorotic phenotypes [18-20], and had lower Ni accumulation under Fe-deficient conditions than the wild type plants. These results indicated that *AtIRT1* mediates Fe and Ni translocation in *Arabidopsis*[21]. Likewise, overexpressing *AtIRT3* leads to increased accumulation of Zn in shoots and Fe in roots. Moreover, AtIRT3 could complement the Zn and Fe uptake double yeast mutants, indicating that AtIRT3 is involved in Zn and Fe translocation [22]. Besides, expression analysis revealed that the transcripts of *AtZIP1* to *AtZIP5*, *AtZIP9* to *AtZIP12*, and *AtIRT3* were increased in response to Zn-deficiency, suggesting that they may enhance Zn acquisition under deficient Zn status in *Arabidopsis*[23]. In rice, overexpression of *OsIRT1* leads to increased Fe and Zn accumulations in shoots, roots and mature seeds, suggesting OsIRT1 is a functional metal transporter for iron, and it is responsible for the absorption of iron from soil, especially under Fe-deficiency [24-26]. On the contrary, over accumulation of *OsZIP4* and *OsZIP5* cannot increase the Zn content in seeds, though the Zn concentration in roots were dramatically increased in transgenic plants [27,28]. These results indicated that maintaining the endogenous expression pattern of *ZIP* genes may be essential for Zn translocation in plants. Likewise, overexpression of *TdZIP1*, a Zn transporter from wild emmer wheat, causes excess accumulation of Zn in cells, thus generating a toxic cytosolic environment [29]. Therefore, increase Zn content by transgenic approaches may benefit from elucidating the expression pattern of *ZIP* genes.

Since *ZIP* is the key transporter for Zn and Fe uptake and translocation in plants, considerable progress has been achieved in cloning and characterizing its functions in crop plants, including soybean and maize [30,31]. The soybean GmZIP1 is highly selective for Zn, and it might play a role in the symbiotic relationship between soybean and *Bradyrhizobium japonicum*[30]. The *ZmZLP1* (ZmZIP-like protein) was identified from a cDNA library of *Zea mays* L. (maize) pollen. It was reported that ZmZLP1 localized to the endoplasmic reticulum and may be responsible for transporting zinc from the ER to the cytoplasm, though its physiological function has not been characterized [31]. The maize genome has been thoroughly sequenced and assembled. However, systematic analysis of the maize *ZIP* gene family is still limited. In the present study, we provide detailed information on the gene identification, chromosomal locations, subcellular localizations and expression patterns of nine *ZmZIP* genes. In addition, the transporter activities of ZmZIPs were tested by yeast complementation analysis. Our results suggest that *ZmZIP* genes may be responsible for the uptake and translocation of Zn or Fe and involve in detoxification and storage of metals in plant cells, as well as embryo and endosperm development.

## Results

### Cloning of *ZmZIP* genes and phylogenetic analysis

**Figure 1:**
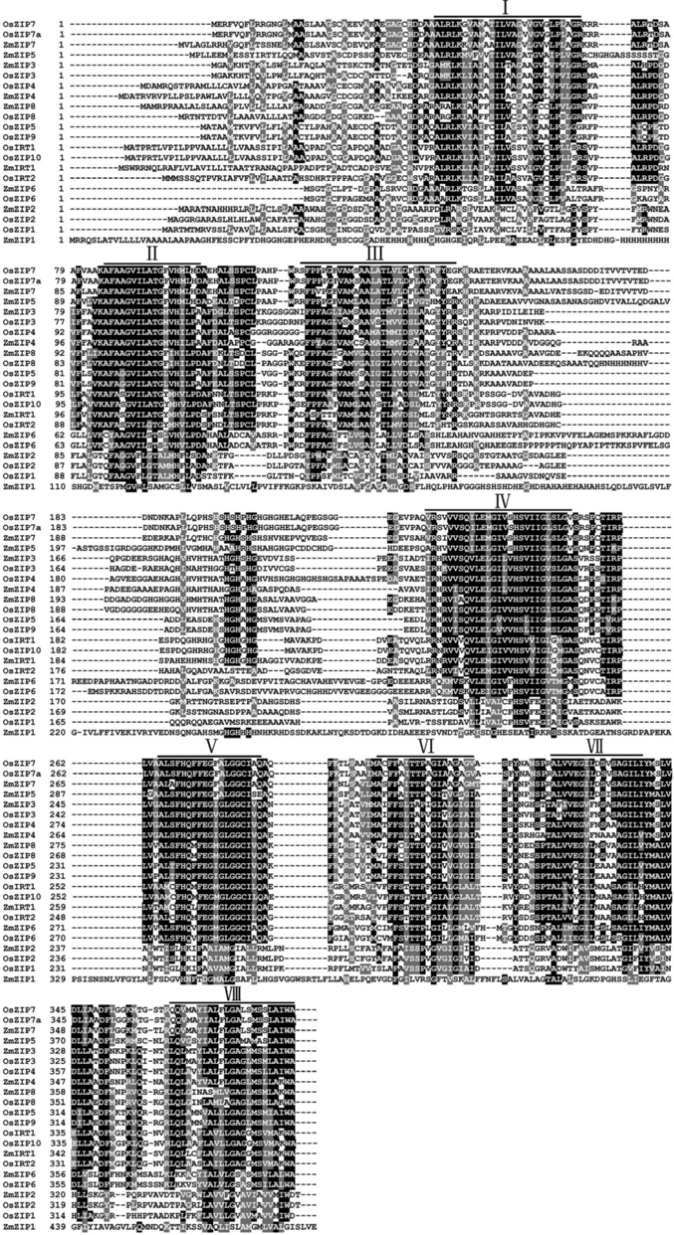
**Amino acid alignment of predicted ZIP proteins in maize genome.** Sequences were aligned using Clustal X Version 2.0 and identical or similar amino acids are shaded by BOXSHADE (http://www.ch.embnet.org/software/BOX_form.html). The membrane spanning domains are indicated as lines above the sequence, and numbered I–VIII, respectively.

**Figure 2:**
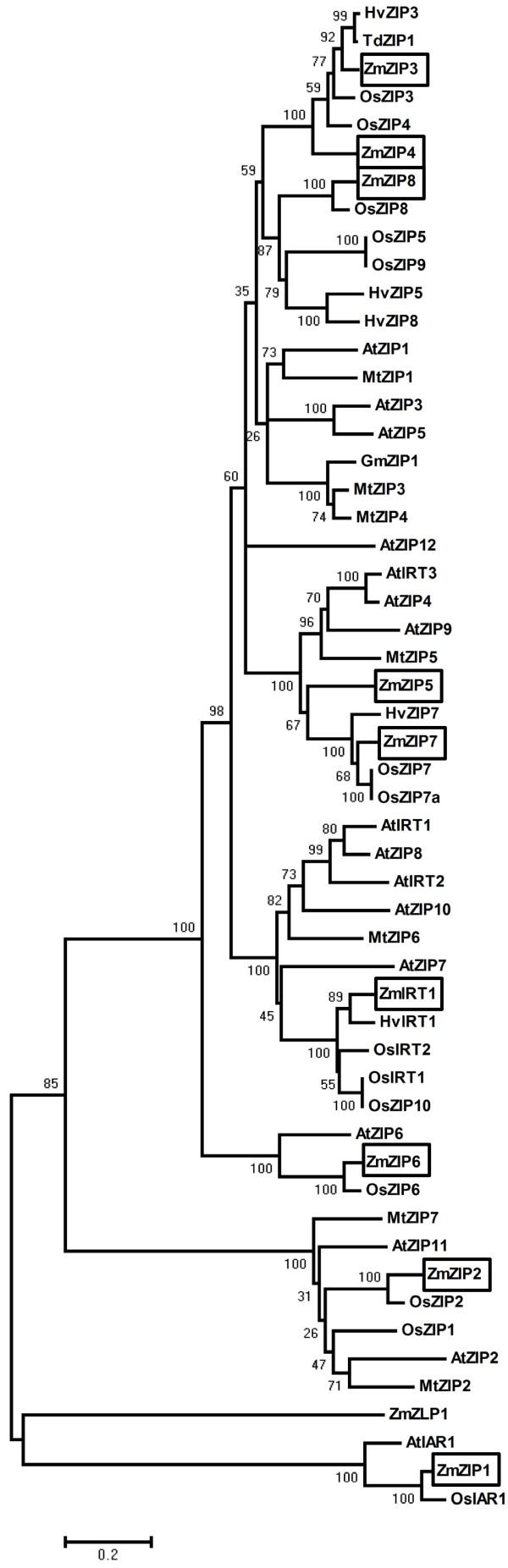
**Phylogenetic analysis of the ZIP family members from various species.** The unrooted phylogenetic tree was constructed with the deduced protein sequences of ZIP proteins from various species using the neighbor-joining method in MEGA 4.0 software. For proteins and accession numbers used for phylogenetic analysis, refer to Methods. The ZmZIP proteins are boxed, and the scale bar corresponds to a distance of 20 changes per 100 amino acid positions.

Extensive searches of public genomic databases, by using reported ZIP proteins from rice as TBLASTN queries, identified a total of nine maize *ZIP* genes that have complete sequences. Nine cDNA fragments containing complete opening reading frame (ORF) were cloned from leaf tissues of maize (*Zea mays* inbred line X178) by RT-PCR, and they were designated as *ZmZIP1-8 and ZmIRT1* according to the amino acid sequence similarity with the rice and *Arabidopsis* (Table 1). The predicted proteins of ZmZIPs consist of 359-490 amino acids, and harbour 6-9 putative transmembrane (TM) domains. In addition, there was a variable region between TM-3 and TM-4, containing a potential metal-binding domain rich in histidine residues (Figure 1). The phylogenetic analysis showed that ZmZIP1 was closely related to AtIAR1 and OsIAR1; ZmIRT1 was related to HvIRT1 and resided in a branch comprised by iron transporter OsIRT1 and OsIRT2. It was suggested that the amino acid residue D100 in AtIRT1 is responsible for Fe and Mn transport [22], and it is conserved in ZmIRT1 (Additional file 1), which indicates that ZmIRT1 may be functional related to a Fe transporter. In addition, ZmZIP3 and ZmZIP4 formed a gene cluster with OsZIP3 and OsZIP4; ZmZIP2 was most closely related to OsZIP2 in the other branch comprised by zinc transporters OsZIP1, AtZIP2 and AtZIP11; ZmZIP5 and ZmZIP7 were observed in a branch; ZmZIP8 and ZmZIP6 were closely related to OsZIP8 and OsZIP6, respectively (Figure 2). These results revealed that ZmZIPs may be functional Zn or Fe transporters.

**Table 1:** **List of *ZIP *genes in the maize genome**

**Gene name**	**Gene ID**^**a**^	**NCBI accession**	**Chromosome**	**CDS length**^**b**^	**Protein length**	**TM domains**
*ZmZIP1*	LOC100192506	NM_001137726	1	1473	490	7
*ZmZIP2*	LOC100286282	NM_001159169	2	1080	359	9
*ZmZIP3*	LOC100282628	NM_001155536	2	1104	367	6
*ZmZIP4*	LOC100280287	HM048832	4	1161	386	6
*ZmZIP5*	LOC100281339	NM_001154257	6	1209	402	6
*ZmZIP6*	LOC100283249	NM_001156151	8	1191	396	8
*ZmZIP7*	LOC100284121	NM_001157018	6	1164	387	6
*ZmZIP8*	LOC100281849	NM_001154769	7	1191	396	7
*ZmIRT1*	LOC100285748	NM_001158638	1	1146	381	8

^a^The Gene ID from MaizeGDB.

^b^CDS of each predicted gene sequence was obtained from Genbank by BLAST search.

### Expression patterns of *ZmZIP* genes in different tissues of maize

**Figure 3:**
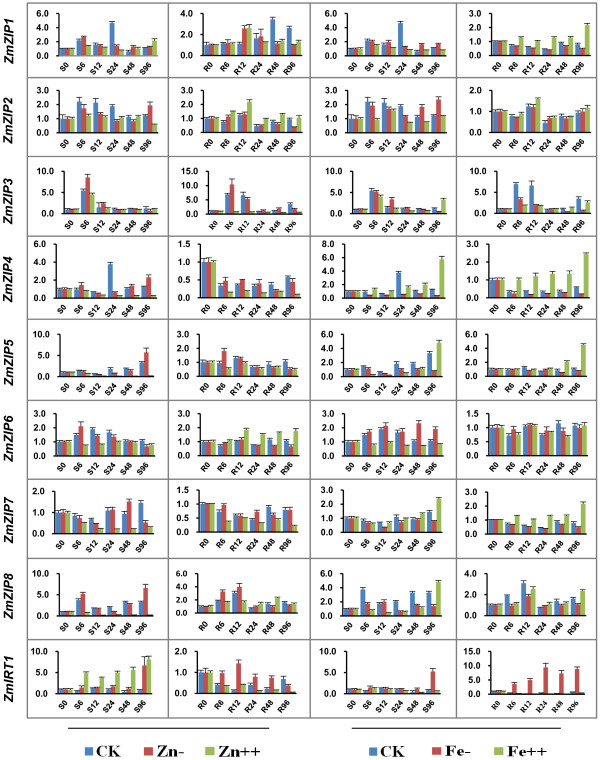
**Expression patterns of the nine *ZmZIP *genes in maize seedlings under various metal conditions.** Two-week old shoots (S) and roots (R) of maize seedlings, under standard nutrient condition (CK), Zn, Fe-deficiency (Zn-), (Fe-), 200 μM ZnSO_4_ (Zn++) and 500 μM FeSO_4_ (Fe++) treated, were harvested respectively at 0 h, 6 h, 12 h, 24 h, 48 h, 96 h after treatment. Relative mRNA abundance of each gene was normalized with *ZmActin1* gene. Data from real-time RT-PCR experiments were analyzed according to the 2^-∆∆Ct^ method. The error bars indicate standard deviations.

Recently, functional analysis has revealed that some *ZIP* genes in *Arabidopsis* and rice play important roles in transporting Zn or Fe [26,27,32]. To better understand the physiological functions of *ZmZIP* genes, their expression patterns in seedlings (under various metal conditions), embryo and endosperm were investigated by real-time RT-PCR. Under sufficient metal nutritional conditions, eight *ZmZIP* genes were mainly expressed in shoots, while *ZmIRT1* was accumulated abundantly in both shoots and roots (Additional file 2). In addition, the expression profiles of *ZmZIP* genes were determined in response to fluctuating divalent metal status. Under Zn-deficient conditions, the expression levels of *ZmIRT1*, *ZmZIP5* and *ZmZIP8* were up-regulated in shoots at 96 h, whereas *ZmZIP3* was induced in both shoots and roots at 6 h (Figure 3). The accumulation of *ZmIRT1* was gradually increased in response to Zn-excess in shoots and reached the maximum level at 96 h after treatment, while that was suppressed in roots. Moreover, the expression of *ZmZIP4, 5, 7* and *8* were suppressed and decreased gradually in shoots, while *ZmZIP3* was down-regulated remarkably in roots (Figure 3). These results indicated that *ZmIRT1*, *ZmZIP3*, *4, 5, 7* and *8* are sensitive to fluctuating environmental Zn status in seedlings.

Under Fe-deficient conditions, the expression of *ZmIRT1* was dramatically up-regulated in shoots and roots. In response to Fe-excess, the transcript level of *ZmIRT1* was suppressed in shoots and roots, while that of *ZmZIP4, 5, 7* and *8* were increased and reached to the maximum level at 96 h in both shoots and roots (Figure 3). These results indicated that *ZmIRT1* and *ZmZIP4, 5, 7, 8* are sensitive to environmental Fe conditions in shoots and roots. Under Cu- and Mn-deficient conditions, the expression patterns of *ZmZIP* genes showed no obvious change (Additional file 3).

**Figure 4:**
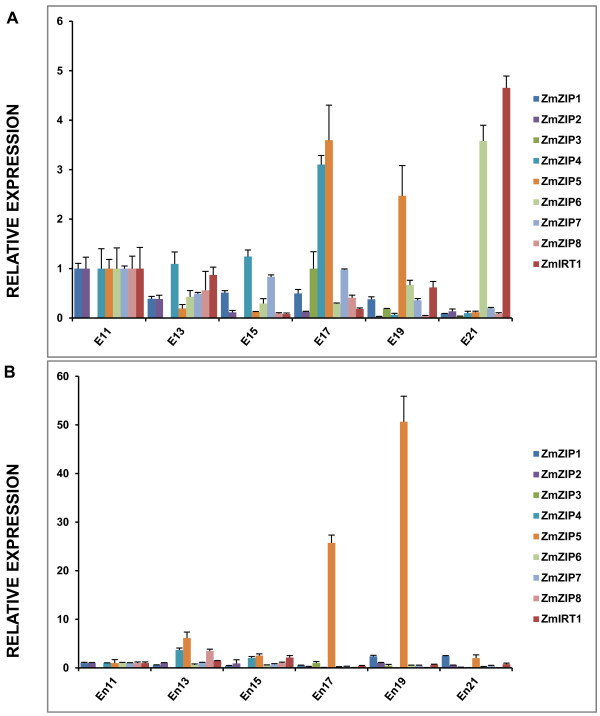
**Expression patterns of the nine *ZmZIP *genes during embryo and endosperm development.** The developing embryo (E) and endosperm (En) were obtained on indicated days after pollination (11 DAP, 13 DAP, 15 DAP, 17 DAP, 19 DAP and 21 DAP). The relative mRNA abundance of each gene in embryo **(A)** and endosperm **(B)** was normalized with *ZmActin1* gene. Data from real-time RT-PCR experiments were analyzed according to the 2^-∆∆Ct^ method. The error bars indicate standard deviations.

A detailed analysis of the transcriptional profiles of *ZmZIP* genes along embryo and endosperm development of maize were measured by real-time RT-PCR. Total RNA was isolated from embryo and endosperm on indicated days after pollination (DAP) (Figure 4A and 4B). The results revealed that the expression levels of *ZmZIP4* and *ZmZIP5* were up-regulated on 17 DAP, while *ZmIRT1* and *ZmZIP6* were mainly expressed in the late developmental stages of embryo. Moreover, the expression of *ZmZIP5* was dramatically increased associated with endosperm development and reached a peak on 19 DAP (approximately 50-fold higher than that of 11 DAP) (Figure 4A and 4B). Taken together, these results suggested that *ZmIRT1*, *ZmZIP4* and *ZmZIP6* may play important roles during embryo development in maize, while *ZmZIP5* may be essential for both embryo and endosperm development.

### Subcellular localization of ZmZIPs

**Figure 5:**
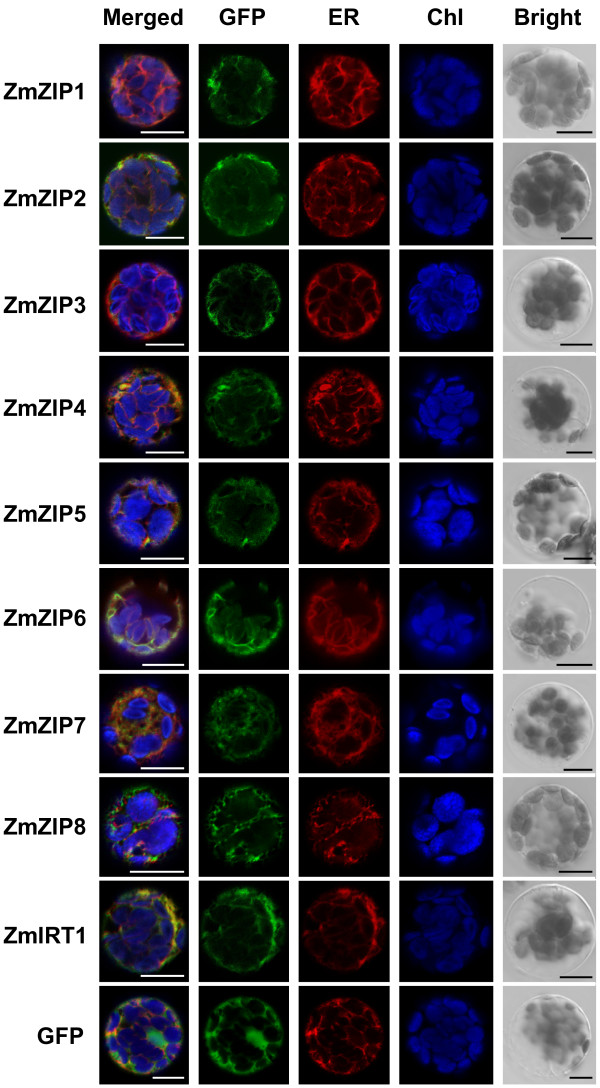
**Subcellular localization of ZmZIPs in *Arabidopsis *mesophyll protoplasts.** Full-length coding regions without stop codon of the *ZmZIP* genes were cloned into the pRTL2GFP vector and the resulting plasmid was transiently transformed into *Arabidopsis* mesophyll protoplasts by the PEG method. The fluorescence of ER marker is indicated in red, the GFP signal is shown in green and the autofluorescence of the chlorophyll (Chl) is artificially stained in blue. The images were obtained by a confocal microscope, and the cytoplasm localization of GFP is used as a control. The scale bar represents 10 μm.

To study the localization of ZmZIP proteins, the full-length coding regions without termination codons of the *ZmZIP* genes were C-terminal fused with green fluorescent protein (GFP) and the fusion proteins were expressed under cauliflower mosaic virus 35S promoter. The ZmZIPs-GFP fusion proteins were transiently expressed in *Arabidopsis* mesophyll protoplasts and onion epidermal cells. The green fluorescence was visualized by confocal microscopy. As showed in Figure 5 and Additional file 4, all ZmZIP proteins were localized to the plasma membrane and endomembrane system, which was further identified as endoplasmic reticulum (ER) by co-localization with an ER marker in *Arabidopsis* mesophyll protoplasts (Figure 5). In addition, the GFP signal can be observed at the border of the cell, indicating the plasma membrane localization of ZmZIPs (Additional file 4). Moreover, plasmolysis was performed to further confirm the plasma membrane localization (Additional file 5). Cells expressing GFP were used as controls, which showed green fluorescence in nucleus and cytoplasm.

### Complementation in yeast cells

**Figure 6:**
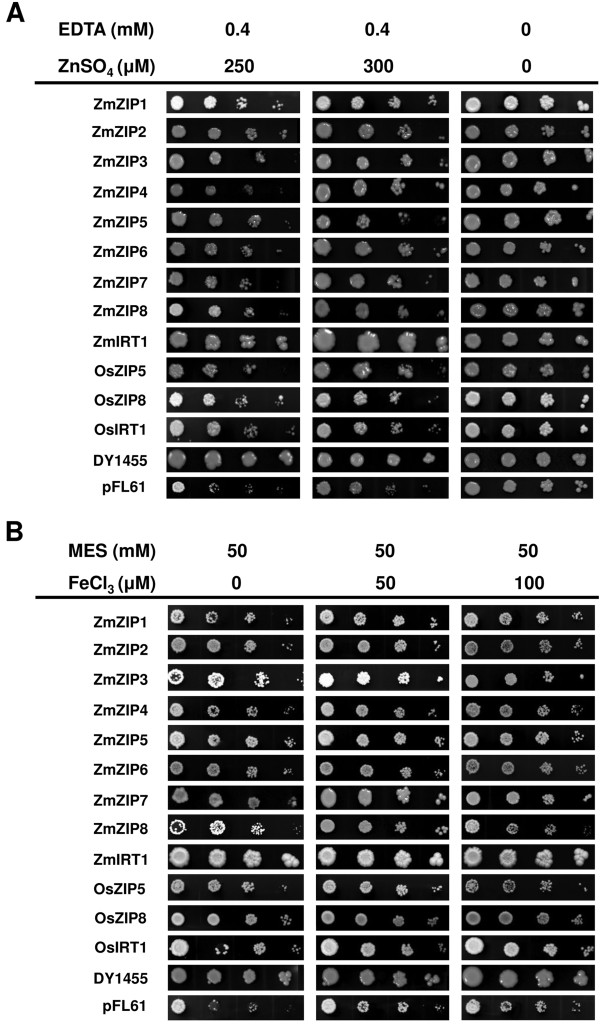
**Functional complementation in yeast mutant strains.** The Zn and Fe transportation mutant *zrt1zrt2***(A)** and *fet3fet4***(B)** were transformed with the expression vector pFL61 carrying *ZmZIP1-ZmZIP8*, *ZmIRT1* and the functional characterized *ZIP* genes, *OsZIP5*, *OsZIP8* and *OsIRT1*. The wild type strain DY1455 transformed with pFL6 was applied as a positive control, and the yeast mutant transformed with empty vector pFL61 was used as a negative control. The transformed yeast cells were grown under different metal conditions as indicated, and the transformed *fet3fet4* was grown on medium with pH 5.8. Cell concentration was adjusted to OD_600_ = 1 and serial dilutions (1.0, 0.1, 0.01 and 0.001) were made. For assay, 5-μL of each dilution was spotted on plates and grown for 6 days at 30°C.

The yeast complementation analysis was performed in order to test whether ZmZIPs possess the capacity of transporting Zn or Fe in organisms. Although, it was demonstrated that Mn is a potential substrate for ZIPs [22,33,34], it has not been tested in this study. The full-length cDNAs of *ZmZIP* genes were expressed in the *Saccharomyces cerevisiae zrt1zrt2* double mutant (ZHY3) and *fet3fet4* double mutant (DEY1453). The transformed ZHY3 expressing *ZmZIP* genes were grew on SD media plus 0.4 mM EDTA. The result showed that the expression of ZmIRT1 protein remarkably reversed this growth defect in the yeast mutant, while other ZmZIPs reversed this growth defect as efficiently as the functional characterized ZIP proteins, OsZIP5 and OsZIP8 (Figure 6A). To test the iron transporter activity, the yeast mutants DEY1453 transformed with pFL61-*ZmZIPs* were grown on SD media plus 50 mM MES. The result revealed that ZmIRT1 reversed the growth defect significantly, while the effect of other proteins were relatively inferior, though that was as efficiently as a reported iron transporter OsIRT1 (Figure 6B). It was interesting to find that the yeast mutants expressing *ZmIRT1* showed the strongest propagation under both Zn- and Fe-limited conditions (Figure 6A and 6B), indicating that *ZmIRT1* may be essential for Zn and Fe translocation during maize growth. In addition, except for ZmIRT1, other ZmZIP proteins can complement the iron transporter mutant *fet3fet4* at high pH but weakly at low pH conditions (Additional file 6). Thus, the *ZmZIP* genes identified in this study encoded functional Zn or Fe transporter, and *ZmIRT1* may play a dominant role in ion translocation in maize.

## Discussion

The *ZIP* genes have been reported in several plants, including *Arabidopsis*, rice, *Medicago truncatula*, wild emmer wheat, *Vitis vinifera* L and barley [18-22,24-29,32,33,35-45]. Most of these genes are found to function as Zn or Fe transporter, though some ZIPs have major roles in Mn transport [22,33,34]. Members of the ZIP family have eight predicted transmembrane (TM) domains and a variable region between TM-3 and TM-4 where contains a potential metal-binding domain [15]. Although ZIPs have been characterized in many plants, to the best of our knowledge, there were few reports concerning the isolation and functional characterization of ZIPs in maize. Since, the maize genome sequencing project was completed, many gene families have been identified and characterized in maize [46-48]. In this study, nine cDNAs encoding *ZmZIP* genes were obtained from maize based on amino acids sequences similarity. In order to analyze the evolutionary relationship of the ZIP family, a phylogenetic tree consists of nine ZIPs and one ZLP1 from maize, 14 rice ZIPs (OsZIPs), 16 *Arabidopsis* ZIPs (AtZIPs), 5 *Hordeum vulgare* ZIPs (HvZIPs), 7 *Medicago truncatula* ZIPs (MtZIPs), Wild Emmer Wheat ZIP (TdZIP1), and *Glycine max ZIP* (GmZIP1) was generated (Figure 2). It was revealed that the predicted amino acid sequences of ZmZIPs were closely related to ZIPs from other plant species and they were existed as orthologs (ZmIRT1 and HvIRT1, ZmZIP1 and OsIAR1, ZmZIP2 and OsZIP2, ZmZIP3 and OsZIP3, ZmZIP4 and OsZIP4, ZmZIP6 and OsZIP6, ZmZIP7 and OsZIP7, ZmZIP8 and OsZIP8), indicating that those ZIPs from maize, rice and *barley* may share a common evolutionary ancestor. It has been reported that OsZIP4, OsZIP5 and OsZIP8 are functional zinc transporters and localized to the plasma membrane [27,32,36]. AtIRT2 is an iron transporter and localized to intracellular vesicles, suggesting an essential role in preventing metal toxicity through compartmentalization and remobilize iron stores from internal storage vesicles [40]. All of these proteins are able to complement the growth of the yeast strain ZHY3 or DEY1453, which is sensitive to Zn or Fe deprivation due to the mutation in both high and low Zn or Fe affinity system. In this study, similar to the *Arabidopsi*s and rice ZIPs, the maize ZIP proteins showed different degree of zinc or iron complementary capabilities (Figure 6A and 6B). Moreover, ZmZIPs were localized to the plasma membrane and endoplasmic reticulum (Figure 5), suggesting they may functional related to excessive ion detoxification. Hence, these results demonstrated that *ZmZIP* genes encode Zn or Fe transporters and have various functions associated with uptake and translocation, detoxification and storage of Zn or Fe in plant cells.

The expression patterns of *ZmZIP* genes reflect their diverse functions during Zn or Fe translocation. It has been reported that the *ZIP* genes displayed various expression profiles regarding tissue specificity and response to fluctuating environmental Zn and Fe conditions. For example, *OsZIP7a* was induced mainly in Fe-deficient roots, while *OsZIP8* was stimulated in Zn-deficient shoots and roots [41]. Histochemical localization analysis showed that the mRNA of *OsZIP4* was accumulated in the vascular bundles of leaves and roots, phloem cells of the stem and the meristems [36]. In the model legume *Medicago truncatula*, the expression of *MtZIP2* was detected in roots and stems and was induced by Zn deficiency [49], likewise *MtZIP1* was expressed in Zn-deficient roots and leaves [37]. In our study, the expression of *ZmIRT1* was remarkably up-regulated in roots and shoots under Fe-deficiency, and was induced in shoots at 96 h after Zn-deficiency (Figure 3). *ZmZIP4*, *5*, *7* and *8* were induced and reached a peak in shoots and roots at 96 h after Fe-excess (Figure 3). These results suggested that *ZmIRT1* may play essential roles in Fe and Zn uptake, especially under iron deficiency, while *ZmZIP4*, *5*, *7* and *8* may associate with detoxification and storage of excessive Fe. Previous study showed that *Arabidopsis thaliana* transcription factors bZIP19 and bZIP23 regulate the adaptation to zinc deficiency by increase the transcription of *ZIPs* and other genes [50]. Therefore, maize bZIP-like transcription factors may be essential for the regulation of *ZmZIP* expression under Zn deficient status.

It has been demonstrated that Zn play essential roles in embryo and endosperm development [2-4]. Therefore, the *ZmZIP* genes that preferentially expressed in embryo and endosperm may be important for translocation of Zn^2+^ into sink organs. The expression analysis showed that *ZmIRT1*, *ZmZIP4* and *ZmZIP6* were mainly expressed in embryo, while *ZmZIP5* was expressed in both embryo and endosperm. The accumulations of *ZmZIP4* and *ZmZIP5* were up-regulated in the middle stages of embryo development and then they were repressed, suggesting that they may be essential for plumule and radicle growth. In contrast, *ZmIRT1* and *ZmZIP6* were stimulated in the late development stages of embryo, which indicates that they may associate with the maturation of embryo. Interestingly, it was observed that the accumulation of *ZmZIP5* was dramatically up-regulated during the development of endosperm and reached a peak on 19 DAP, though its expression was decreased on 21 DAP, which suggests that *ZmZIP5* may involve in the accumulation of nutrient substance at early grain filling stage. By the light of well assembled genome sequence of maize, an *in silico* promoter analysis was performed for those *ZmZIP* genes preferentially expressed in embryo and endosperm. The results showed that the *ZmIRT1*, *ZmZIP4*, *ZmZIP5* and ZmZIP*6* contain *cis*-elements for seed expression (Additional file 7). Considering that there was some correlation between the *VvZIP3* expression profile and the Zn accumulation pattern during the development of reproductive organs [42], we assumed that *ZmIRT1*, *ZmZIP4*, *ZmZIP5* and *ZmZIP6* may play various roles in both embryo and endosperm development.

Enhancing the iron and zinc content in cereal grains is important for improving human nutrition. Since the amount of metal transporter is generally rate-limiting [39], manipulating the transporters involved in translocation of micro-essential metals into sink organs could be a way to increase mineral contents. For instance, overexpressing *AtZIP1* in barley resulted in a rise in the short-term zinc uptake as well as higher seed Zn and Fe contents [39]. Likewise, the iron and zinc contents were elevated in the shoots, roots and mature seeds of transgenic rice constitutively overexpressing *OsIRT1*[26]. However, overexpressing *OsZIP4* under the control of the cauliflower mosaic virus (CaMV) 35S promoter lead to Zn accumulation in roots, while the Zn concentration in seeds were four times lower than untransgenic controls [28]. Moreover, overexpressing *OsZIP5* and *OsZIP8* in rice under the control of the maize *ubiquitin* promoter lead to increased Zn level in roots, though that in shoots and mature seeds were reduced in the transgenic plants [27,32]. These results indicate that ectopic overexpression of ZIP proteins may have little effect on the enhancement of Zn content in seeds due to overproduction of ZIP in vegetative tissues. Therefore, increasing the accumulation of ZIP proteins and maintain their expression pattern may provide an alternative way to enhance Zn or Fe contents. Since it was revealed that the expression of *ZmIRT1*, *ZmZIP4*, *ZmZIP5* and *ZmZIP6* are associated with seed development, it can be assumed that overexpression of these *ZIP* genes in a seed specific manner may provide an alternative strategy for biofortification of crops with Zn and Fe.

## Conclusions

Although zinc and iron are essential micronutrients for plant growth and development, functional analysis of ZIP family in maize is still limited. The present study provides relevant information concerning the identification and functional characterization of ZmZIP family, and suggests that they may involve in metal uptake and overall cell zinc homeostasis*.* It is also indicated that *ZmZIPs* may be essential for ion translocation and storage during differential stages of embryo and endosperm development. In the present study, we provided detailed information of the evolutionary relationship and putative functional divergence of the *ZmZIP* gene family during the growth and development of maize.

## Methods

### Plant growth

*Zea mays* seeds were cultured in vermiculite (irrigated with Hoagland nutrient solution) in climate chambers with a light/dark cycle of 16/8 h. The 13-day-old seedlings were transferred to the Hoagland nutrient solution grown for 6 days (The standard Hoagland nutrient solution contained 2.0 μM ZnSO_4_, 50 μM Fe(III)-EDTA, 0.5 μM CuSO_4_, and 2.0 μM MnSO_4_), then transferred to the standard Hoagland nutrient solution and Hoagland medium lacking ZnSO_4_ (Zn-deficient), Fe (III)-EDTA (Fe- deficient), and 200 μM ZnSO_4_ (Zn-excess), 500 μM Fe(III)-EDTA (Fe-excess). Shoots and roots were selected and detached at 0 h, 6 h, 12 h, 24 h, 48 h and 96 h after the different treatment for real-time RT-PCR assays.

### Bioinformatics analysis

The BLAST program at GenBank (http://www.ncbi.nlm.nih.gov/blast) was used to search the maize ZIP cDNAs and the acquired cDNAs were compared to the corresponding genome database from maizesequence (http://maizesequence.org/index.html). The putative amino acid sequences were aligned with the program Clustal X Version 2.0 [51] and colored by BOXSHADE (http://www.ch.embnet.org/software/BOX_form.html). Potential transmembrane domains in protein sequences were identified using TMHMM [52]. The phylogenetic tree of 54 members of the ZIP proteins from various species was constructed using MEGA version 4.0 [53]. The accession numbers for the proteins are as follows: *Arabidopsis thaliana* (AtZIP1: AAC24197, AtZIP2: AAC24198, AtZIP3: AAC24199, AtZIP4: AAB65480, AtZIP5: AAL38432, AtZIP6: AAL38433, AtZIP7: AAL38434, AtZIP8: AAL83293, AtZIP9: AAL38435, AtZIP10: AAL38436, AtZIP11: AAL67953, AtZIP12: AAL38437, AtIRT1: AAB01678, AtIRT2: NP_001031670, AtIRT3: NP_564766, AtIAR1: AF216524); *Oryza sativa* (OsIAR1: NP_001062003, OsIRT1: AB070226, OsIRT2: AB126086, OsZIP1: AY302058, OsZIP2: AY302059, OsZIP3: AY323915, OsZIP4: AB126089, OsZIP5: AB126087, OsZIP6: AB126088, OsZIP7: AB126090, OsZIP7a: AY275180, OsZIP8: AY324148, OsZIP9: AY281300, OsZIP10: AK107681); *Zea mays* (ZmZIP1: NM_001137726, ZmZIP2: NM_001159169, ZmZIP3: NM_001155536, ZmZIP4: HM048832, ZmZIP5: NM_001154257, ZmZIP6: NM_001156151, ZmZIP7: NM_001157018, ZmZIP8: NM_001154769, ZmIRT1: NM_001158638, ZmZLP1: ACO50388); *Hordeum vulgare* (HvIRT1: EU545802, HvZIP3: FJ208991, HvZIP5: FJ208992, HvZIP7: AM182059, HvZIP8: FJ208993); *Medicago truncatula* (MtZIP1: AY339054, MtZIP2: AY007281, MtZIP3: AY339055, MtZIP4: AY339056, MtZIP5: AY339057, MtZIP6: AY339058, MtZIP7: AY339059); Wild Emmer Wheat (TdZIP1:AY864924); *Glycine max* (GmZIP1: AY029321).

### RNA isolation and real-time RT-PCR

Total RNA was isolated from shoots and roots with TRIzol (Takara). For cDNA synthesis, we used 4 μg of total RNA as a template and M-MLV reverse transcriptase (Fermentas) by primering with oligo-d(T)_18_ in a 40-μL reaction mixture. Real-time RT-PCR was performed in a 20-μL solution containing a 5-μL aliquot of the cDNA, 0.4 μM of gene-specific primers (Additional file 8) and 10-μL SYBR Green I (Takara). The fragment was amplified by PCR in an ABI 7500 Real Time Thermal Cycler. The constitutively expressed *ZmActin1* gene [GenBank: J01238.1] was amplified as the reference gene using the primers ZmActin1F and ZmActin1R (Additional file 8). Changes in expression were calculated via the ∆∆Ct method [54]. For all real-time RT-PCR analysis, two biological replicates were used and three technical replicates were performed for each biological replicate. The sizes of the amplified fragments were confirmed by gel electrophoresis.

### Cloning of target genes

The cDNA sequences of nine putative maize *ZIP* genes with a complete ORF were obtained from the MaizeSequence database (http://www.maizesequence.org/). The primers (Additional file 8) were designed for amplifying the ORFs of these nine *ZmZIP* genes. Total RNA was isolated from shoots with TRIzol (Takara), and was treated with DNaseI (NEB) before being reverse transcribed. For cDNA synthesis, we used 2 μg of total RNA as a template and M-MLV reverse transcriptase (Fermentas) by primering with oligo-d(T)_18_ in a 20-μL reaction mixture. PCR was performed in a 20-μL solution containing a 5-μL aliquot of the cDNA, 10 μM of gene-specific primers (Additional file 8), 0.5 U Ex Taq polymerase (Takara), 8 mM dNTPs, 10-μL of 2 × GCI buffer. PCR was performed on a DNA amplification machine (BIO RAD) for a denaturation of 4 min at 94°C, followed by 33 cycles of 1 min at 94°C, 1 min at 60°C, and 1 min at 72°C and a final extension of 10 min at 72°C. The PCR products were separated on a 1% agarose gel and purified with Gel DNA Purification Kit (Shenergy Biocolor) according to the manufacturer’s instruction. The purified product was then cloned into the pGEM-T easy vector (Promega, USA) and sequenced (Openlab, China).

### Subcellular localization

For subcellular localization, a C-terminal GFP fusion expression vector pRTL2GFP was used [48]. Gene-specific primers were designed and the stop codons were deleted (Additional file 8). The coding regions without the stop codon were cloned into pRTL2GFP, respectively. The plasmids were purified using the Wizard Plus Miniprep DNA Purification System (Promega). The ZmZIP-GFP fusion constructs and the mcherry labeled ER marker were co-transformed into *Arabidopsis* mesophyll protoplasts as described previously [55,56]. After incubation in the dark at 26°C for 14 h, the fluorescence was examined using a confocal microscope (LSM700; Carl Zeiss). The GFP signal was excitated at 488 nm, and the emission was collected at 500-530 nm; the mcherry signal was excitated at 555 nm, and the fluorescence emission was collected at 610 nm; the 630 emission filter was used to observe the autofluorescence of chlorophyll.

### Yeast complementation

The ORFs of *ZmZIP* genes were amplified with gene specific primers (Additional file 8), and the PCR fragments were purified from an agarose gel and subsequently ligated into the *NotI* site of the yeast expression vector pFL61 (provided from Dr. David Eide, University of Missouri-Columbia) [57]. The resulting constructs were sequenced to ensure the correct orientations of the inserts and sequences. The yeast strains used in this experiment are *zrt1zrt2* ZHY3 (MATα *ade6 can1 his3 leu2 trp1 ura3 zrt1::LEU2 zrt2::HIS3*), *fet3fet4* DEY1453 (*MATa/MATa* ade2/+ *can1/can1 his3/his3 leu2/leu2 trp1/trp1 ura3/ura3 fet3-2::HIS3/fet3-2::HIS3 fet4-1::LEU2/fet4-1::LEU2*), and DY1455 (*MATa ade6 can1 his3 leu2 trp1 ura3*) (provided by Dr. David Eide) [58,59]. The pFL61-*ZmZIPs* were transformed into the yeast strains ZHY3 and DEY1453 using the electroporation method. The empty vector pFL61was applied as a negative control, OsIRT1 was used as the positive control for iron transporter, OsZIP5 and OsZIP8 were applied as the positive control for zinc transporter, and the wild type strain DY1455 harbouring pFL61 was used as another positive control. The yeast complementation was performed as described previously [22] with slight modification. Transformed cells were selected on synthetic agar medium (SD) containing amino acid supplements without Uracil and 2% glucose. In growth-test experiments, 5-μL drops of yeast culture at an optical density of 1.0, 0.1, 0.01 and 0.001 were spotted onto medium. The yeast strain of *zrt1zrt2* ZHY3 were grown on SD/–ura medium (pH 4.4) supplemented with 0.4 mM EDTA and 250 μM or 300 μM ZnSO_4_. The yeast strain of *fet3fet4* were grown on SD/–ura medium (pH 5.5, 5.8 containing 50 mM 2-(N-morpholino) ethanesulfonic acid (MES) supplemented with 0, 50 or 100 μM FeCl_3_.

## Abbreviations

CaMV: Cauliflower mosaic virus; GFP: Green fluorescent protein; ER: Endoplasmic reticulum; ORF: Opening reading frame; RT: Reverse transcripts; TM: Transmembrane; ZIP: Zinc-regulated transporters, iron-regulated transporter-like protein.

## Competing interests

The authors declare that they have no competing interests.

## Authors’ contributions

SZL and JTC participated in the design of the study. SZL performed the bioinformatics analysis, gene cloning, real-time RT-PCR and yeast complementation. XJZ analyzed the data and drafted the manuscript. YQH assisted in gene cloning and plasmid construction. LYZ and SJZ collected the tissues for temporal and spatial expression analysis. YFZ and JJG helped in bioinformatics analysis. JTC and RMC contributed to revisions of the manuscript. All authors read and approved the final manuscript.

## Supplementary Material

Additional file 1 **Amino acid alignment of ZmIRT1 with other IRT proteins.** Sequences were aligned using Clustal X Version 2.0 and the identical or similar amino acids are shaded by BOXSHADE (http://www.ch.embnet.org/software/BOX_form.html). The residue D100 in ZmIRT1 is marked with an asterisk. Click here for file

Additional file 2 **Expression patterns of the nine *ZmZIP *genes in maize seedlings.** The shoots (S) and roots (R) of two weeks old hydroponically cultured maize seedlings in Hoagland nutrient solution were harvested, respectively. Relative mRNA abundance of each gene was normalized with *ZmActin1* gene. Data from real-time RT-PCR experiments were analyzed according to the 2^-∆∆Ct^ method. The error bars indicate standard deviations. Click here for file

Additional file 3 **Expression patterns of the nine *ZmZIP *genes in maize under Cu-, Mn- deficiency.** Two-week old shoots (S) and roots (R) of maize seedlings, under Cu-, Mn-deficiency treated (Cu-), (Mn-) were harvested respectively at 0 h, 6 h, 12 h, 24 h, 48 h and 96 h. Relative mRNA abundance of each gene was normalized with *ZmActin1* gene. Data from real-time RT-PCR experiments were analyzed according to the 2^-∆∆Ct^ method. The error bars indicate standard deviations. Click here for file

Additional file 4 **Subcellular localization of ZmZIPs in onion epidermal cells.** Full-length coding regions without stop codon of the *ZmZIP* genes were cloned into the pRTL2GFP vector and the resulting plasmid was transiently transformed into onion epidermal cells by bombardment. The fluorescence was observed using a confocal laser scanning microscopy. GFP was imaged using 488 nm excitation and a 500-530 nm bandpass emission filter. The scale bar represents 100 μm. Click here for file

Additional file 5 **Subcellular localization of ZmZIP-GFP fusion proteins in plasmolyzed onion epidermal cells.** The ZmZIP-GFP fusion proteins were transiently expressed in onion epidermal cells by bombardment, and a set of representative images are shown. The plasmolysis was performed for 15 min in 30% sucrose. The Z-stack of optical sections and single optical slice of GFP fluorescence are shown. The cytoplasm localization of GFP is used as a control. GFP was imaged using 488 nm excitation and a 500-530 nm bandpass emission filter. The scale bar represents 100 μm. Click here for file

Additional file 6 **Functional complementation of the Fe transportation yeast mutant by *ZmZIPs *under different pH values.** The Fe transportation mutant *fet3fet4* were transformed with the expression vector pFL61 carrying *ZmZIP1-ZmZIP8*, *ZmIRT1* and the functional characterized *ZIP* genes, *OsZIP5*, *OsZIP8*, and *OsIRT1*. The transformed yeast cells were grown under different pH conditions (A) pH 5.5 and (B) pH 5.8. Cell concentration was adjusted to OD_600_ = 1 and serial dilutions (1.0, 0.1, 0.01 and 0.001) were made. For assay, 5-μL of each dilution was spotted on plates and grown for 6 days at 30°C. Click here for file

Additional file 7 **Putative *cis*-elements in the 2-Kb upstream promoter region of translation start site in *ZmZIP *genes.** Click here for file

Additional file 8 Primers used in gene cloning, vector construction and real-time RT-PCR analysis. Click here for file
