# Supplementary material for: Identification and characterization of the zinc-regulated transporters, iron-regulated transporter-like protein (ZIP) gene family in maize
Source: BMC Plant Biol. 2013 Aug 8;13:114. doi: 10.1186/1471-2229-13-114 (PMC3751942; doi:10.1186/1471-2229-13-114)
Supplement: Additional file 1 — Amino acid alignment of ZmIRT1 with other IRT proteins. Sequences were aligned using Clustal X Version 2.0 and the identical or similar amino acids are shaded by BOXSHADE (http://www.ch.embnet.org/software/BOX_form.html). The residue D100 in ZmIRT1 is marked with an asterisk. [file 1471-2229-13-114-S1.pdf]

```
AtIRT1    1 ---------------MKTIFLVLIFVSFAISPATSTAPEECG-----SESANPCVNKAKA
AtIRT2    1 ------------MATTKLVYILLILFTFTVSPAISTAPEHCD-----SGFDNPCINKAKA
OsIRT1    1 --MATPRTLVPILPPVAALLLLLVAASSIPILAAAQPADACGGAPDQAAADGACHDVPRA
OsIRT2    1 -----MMMSSSQTPVRIAFVFLVILAATDAHSDHRTPPPACG----GAAVGGECHSVARA
ZmIRT1    1 -MSWRRNQLRAFLVLAVILLLITAATYRANAQPAPPADPTPAADTCADPSVEGACRNVPKA
AtIRT3    1 MFFVDVLWKLVPLYLFGSETKSLSATESILQIVPEAMAATSSNVLCNASESDLCRDDSAA

                                                                       *
AtIRT1   41 LPLKVIAIFVILIASMIGVGAPLFSRNVSFLQPDGNIFTIIKCFASGIILGTGFMHVLPD
AtIRT2   44 LPLKIVAIVAILTTSLIGVTSPLFSRYISFLRPDGNGFMIVKCFSSGIILGTGFMHVLPD
OsIRT1   59 LRLKLIAIPTILVSSVVGVCLPLLSRSVPALRPDGGLFAVVKAFASGVILATGYMHVLPD
OsIRT2   52 LRLKLIAIPAILAASVAGVCLPLFARSVPALRPDGGLFAVVKAFASGVILGTGYMHVLPD
ZmIRT1   60 LRLKLIAIPTILVSSVIGVCLPLLSRSVPALRPDRNLFVIVKAFASGVILATGYMHVLPD
AtIRT3   61 FLLKFVAIASILLAGAAGVTIPLIGRNRRFLQTDGNLFVTAKAFAAGVILATGFVHMLAG

AtIRT1  101 SFEMLSSICLEENPWHKFPFSGFLAMLSGLITLAIDSMATSLYTSK---NAVGIMP----
AtIRT2  104 SFEMLSSKCLSDNPWHKFPFAGFVAMMSGLVTLAIDSITTSLYTGK---NSVGPVPDE--
OsIRT1  119 AFNNLTSPCLPRKPWSEFPFAAFVAMLAAVSTLMADSLMLTYYNRSKPRPSSGGDVA---
OsIRT2  112 SFNDLTSPCLPRKPWSEFPFAAFVAMLAAVFTLMVDSLMLTFHTRG----SKGRASS---
ZmIRT1  120 SFSNLTSPCLPRKPWADFSFTTFVAMLAALFTLMVDSLMLSFYNRRKGGNTSGRRTSG--
AtIRT3  121 GTEALKNPCLPDFPWSKFPFPGFFAMIAALITLFVDFMGTQYYERKQEREASESVEPFGR

AtIRT1  154 ------------------------------------------------------------
AtIRT2  159 ---------------------------------------------------EYGIDQEKA
OsIRT1  176 ---------------------------------------------------AVADHGESP
OsIRT2  165 ---------------------------------------------------AVAHHG---
ZmIRT1  178 ---------------------------------------------------AVADHESPA
AtIRT3  181 EQSPGIVVPMIGEGTNDGKVFGEEDSGGIHIVGIHAHAAHHRHSHPPGHDSCEGHSKIDI

AtIRT1  154 -HGHGHGHGPANDVTLPIKEDDSSN-------------AQLLRYRVIAMVLELGIIVHSV
AtIRT2  168 IHMVGHNHSHGHGVVLATKDD-----------------GQLLRYQVIAMVLEVGILFHSV
OsIRT1  185 DQGHRHGHGHGHGHGMAVAK----------PDDVEATQVQLRRNRVVVQVLEIGIVVHSV
OsIRT2  171 DHGHCHAHALGQADVAALSTTEAADQGSGDVEAGNTTKAQLLRNRVIVQVLEMGIVVHSV
ZmIRT1  187 HEHHWHSHGHGHGHGHAGGIVVADK-----PEDDEASQVQLRRNRVVVQVLEMGIVVHSV
AtIRT3  241 GHAHAHGHGHGHGHGHVHGGLDAVN---------------GARHIVVSQVLELGIVSHSI

AtIRT1  200 VIGLSLGATSDTCTIKGLIAALCFHQMFEGMGLGGCILQAEYTNMKKFVMAFFFAVTTPF
AtIRT2  211 VIGLSLGATNDSCTIKGLIIALCFHHLFEGIGLGGCILQADFTNVKKFLMAFFFTGTTPC
OsIRT1  235 VIGLGMGASQNVCTIRPLVAAMCFHQMFEGMGLGGCILQAEYGRRMRSVIVFFFSTTTPF
OsIRT2  231 VIGLGMGASQNVCTIRPLVAALCFHQMFEGMGLGGCILQAGYGGRTRSALVFFFSTTTPF
ZmIRT1  242 VIGLGMGASQNVCTIRPLVTAMCFHQLFEGMGLGGCILQAEYGAKMKAGIVFFFSTTTPF
AtIRT3  286 IIGLSLGVSQSPCTIRPLIAALSFHQFFEGFALGGCISQAQFRNKSATIMACFFALTTPI

AtIRT1  260 GIALGIALSTVYQDNSPKALITVGLLNACSAGLLIYMALVDLLAAEFMGPKLQGSIKMQF
AtIRT2  271 GIFLGIALSSIYRDNSPTALITIGLLNACSAGMLIYMALVDLLATEFMGSMLQGSIKLQI
OsIRT1  295 GIALGLALTRVYRDNSPTALIVVGLLNAASAGLLHYMALVELLAADFMGPKLQGNVRLQL
OsIRT2  291 GIALGLALTRVYSDSSPTALVVVGLLNAASAGLLHYMALVELLAADFMGPKLQGNVRLQL
ZmIRT1  302 GIALGLALTKVYRENSPTALIVVGLLNAASAGLLHYMALVELLAADFMGPKLQSSVRLQL
AtIRT3  346 GIGIGTAVASSFNSHSVGALVTEGILDSLSAGILVYMALVDLIAADFLSTKMRCNFRLQI

AtIRT1  320 KCLIAALLGCGGMSIIAKWA
AtIRT2  331 KCFTAALLGCAVMSVVAVWA
OsIRT1  355 AAFLAVLLGAGGMSVMAKWA
OsIRT2  351 AASLAILLGAGGMSVMAKWA
ZmIRT1  362 LCFLAVLLGAGGMSIMAKWA
AtIRT3  406 VSYVMLFLGAGLMSSLAIWA
```
