# Supplementary figures and images for: Identification and characterization of the zinc-regulated transporters, iron-regulated transporter-like protein (ZIP) gene family in maize
Source: BMC Plant Biol. 2013 Aug 8;13:114. doi: 10.1186/1471-2229-13-114 (PMC3751942; doi:10.1186/1471-2229-13-114)

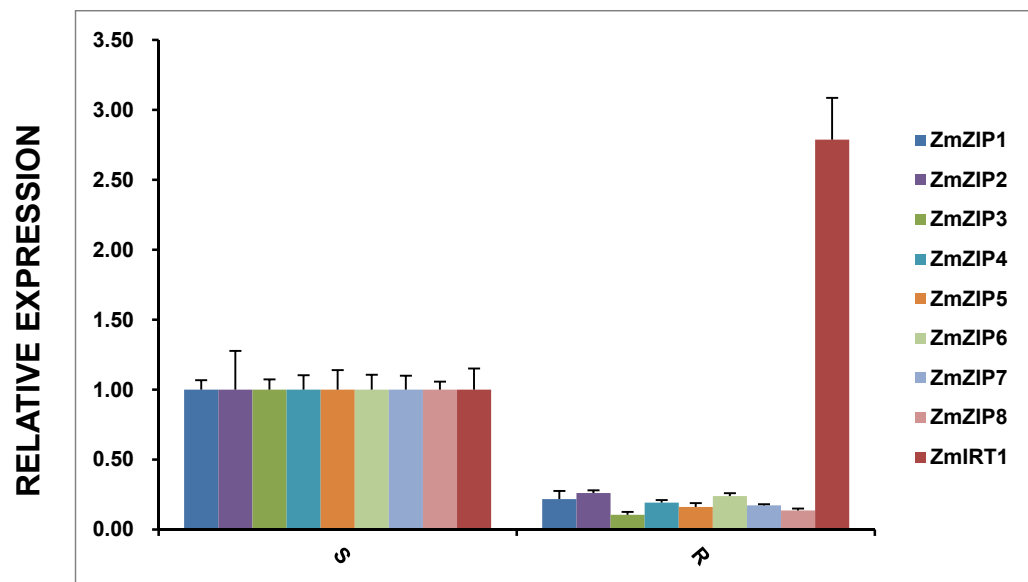

Supplement: Additional file 2 — Expression patterns of the nine ZmZIP genes in maize seedlings. The shoots (S) and roots (R) of two weeks old hydroponically cultured maize seedlings in Hoagland nutrient solution were harvested, respectively. Relative mRNA abundance of each gene was normalized with ZmActin1 gene. Data from real-time RT-PCR experiments were analyzed according to the 2-∆∆Ct method. The error bars indicate standard deviations. [file 1471-2229-13-114-S2.pdf]

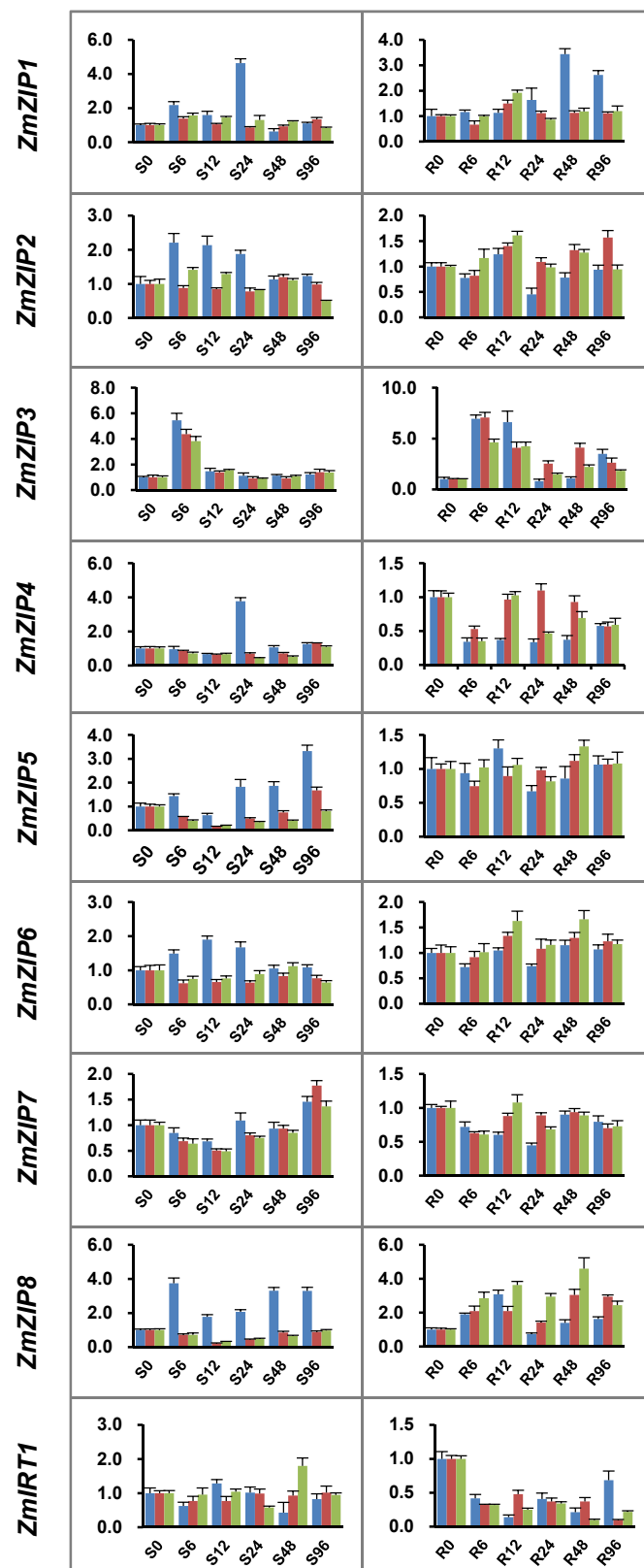

■ CK ■ Cu- ■ Mn-

Supplement: Additional file 3 — Expression patterns of the nine ZmZIP genes in maize under Cu-, Mn- deficiency. Two-week old shoots (S) and roots (R) of maize seedlings, under Cu-, Mn-deficiency treated (Cu-), (Mn-) were harvested respectively at 0 h, 6 h, 12 h, 24 h, 48 h and 96 h. Relative mRNA abundance of each gene was normalized with ZmActin1 gene. Data from real-time RT-PCR experiments were analyzed according to the 2-∆∆Ct method. The error bars indicate standard deviations. [file 1471-2229-13-114-S3.pdf]

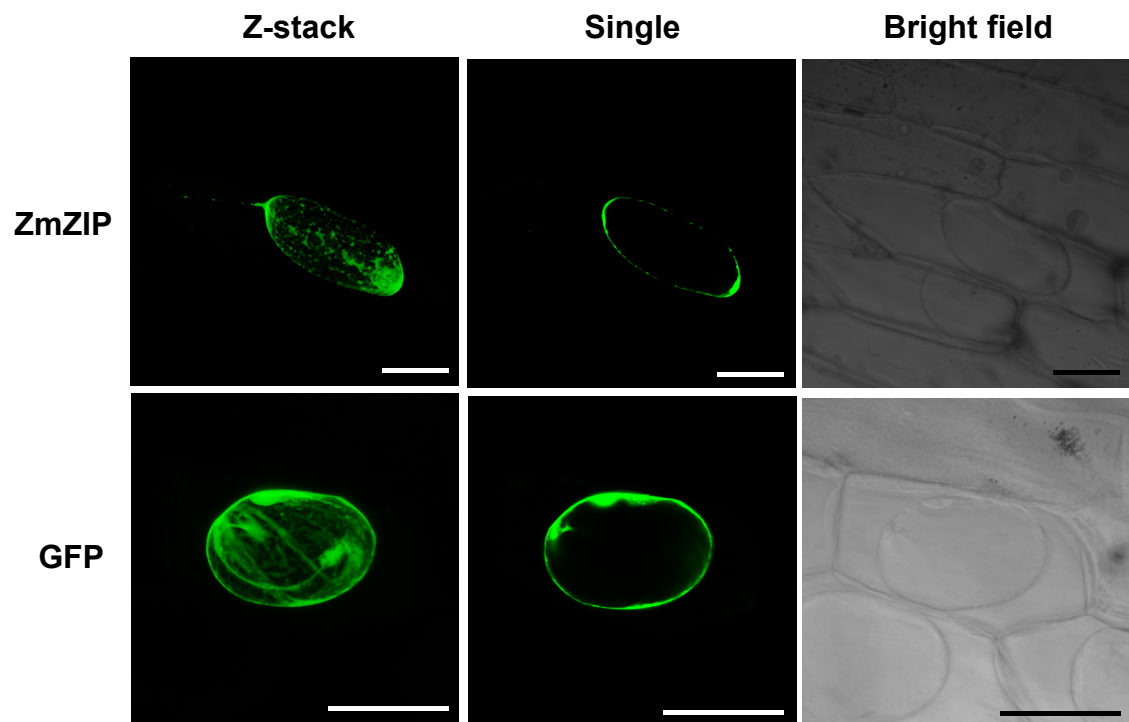

Supplement: Additional file 5 — Subcellular localization of ZmZIP-GFP fusion proteins in plasmolyzed onion epidermal cells. The ZmZIP-GFP fusion proteins were transiently expressed in onion epidermal cells by bombardment, and a set of representative images are shown. The plasmolysis was performed for 15 min in 30% sucrose. The Z-stack of optical sections and single optical slice of GFP fluorescence are shown. The cytoplasm localization of GFP is used as a control. GFP was imaged using 488 nm excitation and a 500-530 nm bandpass emission filter. The scale bar represents 100 μm. [file 1471-2229-13-114-S5.pdf]
