# Supplementary material for: Identification and characterization of the zinc-regulated transporters, iron-regulated transporter-like protein (ZIP) gene family in maize
Source: BMC Plant Biol. 2013 Aug 8;13:114. doi: 10.1186/1471-2229-13-114 (PMC3751942; doi:10.1186/1471-2229-13-114)
Supplement: Additional file 4 — Subcellular localization of ZmZIPs in onion epidermal cells. Full-length coding regions without stop codon of the ZmZIP genes were cloned into the pRTL2GFP vector and the resulting plasmid was transiently transformed into onion epidermal cells by bombardment. The fluorescence was observed using a confocal laser scanning microscopy. GFP was imaged using 488 nm excitation and a 500-530 nm bandpass emission filter. The scale bar represents 100 μm. [file 1471-2229-13-114-S4.pptx]

## Slide 1
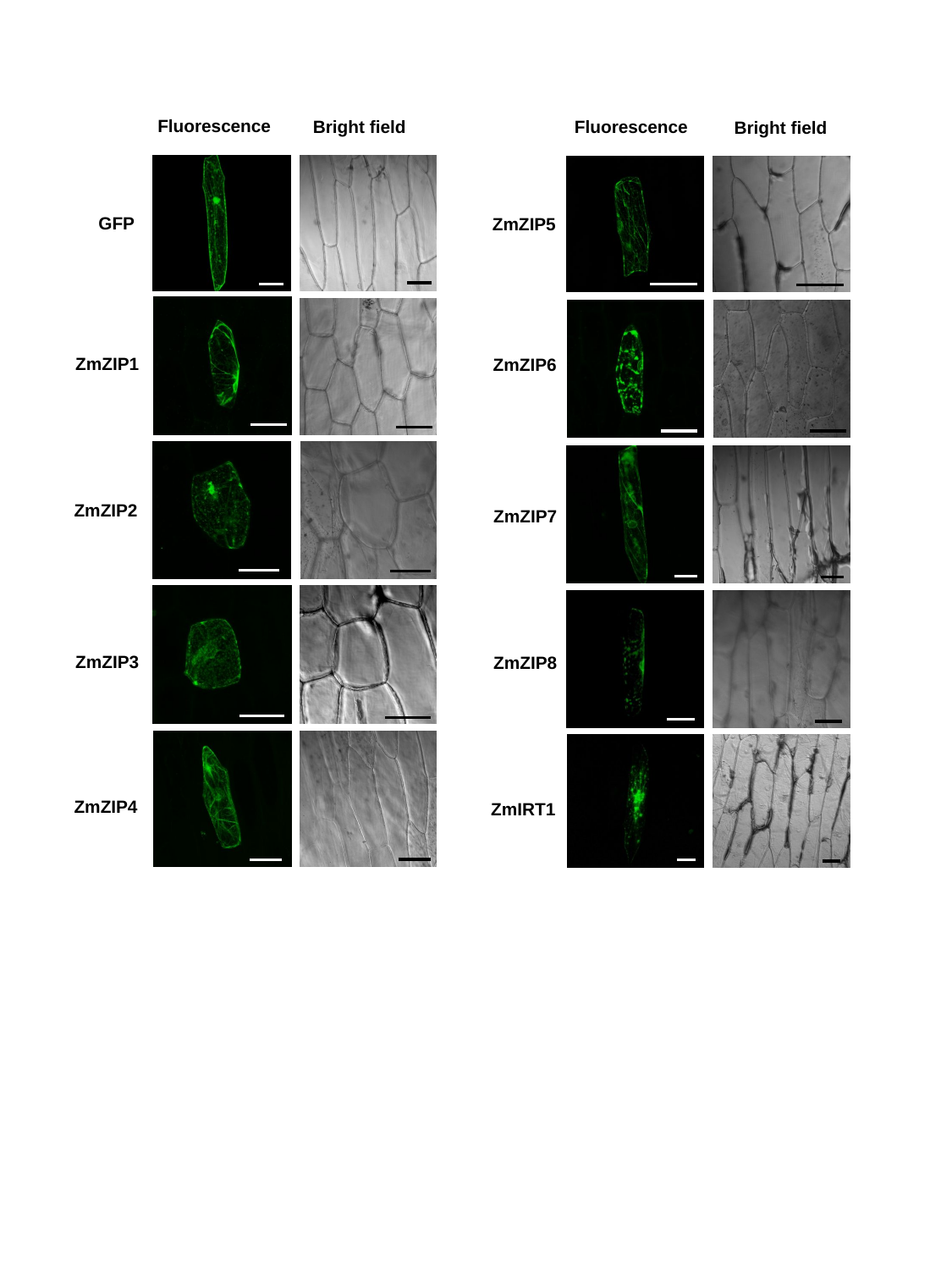

Fluorescence
Bright field
Fluorescence
Bright field
GFP
ZmZIP5
ZmZIP1
ZmZIP6
ZmZIP2
ZmZIP7
ZmZIP3
ZmZIP8
ZmZIP4
ZmIRT1
