# Supplementary material for: Identification and characterization of the zinc-regulated transporters, iron-regulated transporter-like protein (ZIP) gene family in maize
Source: BMC Plant Biol. 2013 Aug 8;13:114. doi: 10.1186/1471-2229-13-114 (PMC3751942; doi:10.1186/1471-2229-13-114)
Supplement: Additional file 6 — Functional complementation of the Fe transportation yeast mutant by ZmZIPs under different pH values. The Fe transportation mutant fet3fet4 were transformed with the expression vector pFL61 carrying ZmZIP1-ZmZIP8, ZmIRT1 and the functional characterized ZIP genes, OsZIP5, OsZIP8, and OsIRT1. The transformed yeast cells were grown under different pH conditions (A) pH 5.5 and (B) pH 5.8. Cell concentration was adjusted to OD600 = 1 and serial dilutions (1.0, 0.1, 0.01 and 0.001) were made. For assay, 5-μL of each dilution was spotted on plates and grown for 6 days at 30°C. [file 1471-2229-13-114-S6.pptx]

## Slide 1
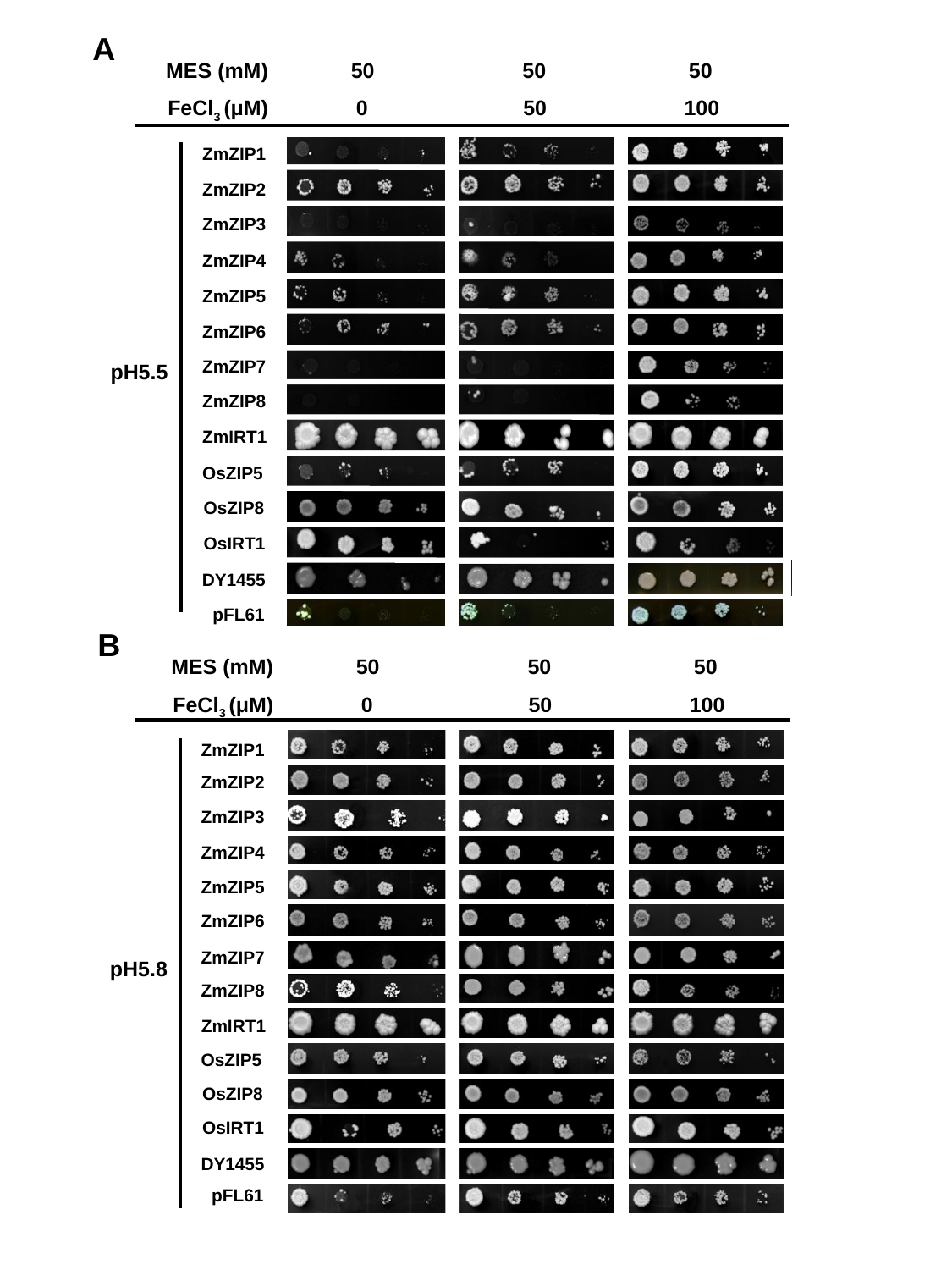

A
MES (mM)
50
50
50
FeCl3 (μM)
0
50
100
ZmZIP1
ZmZIP2
ZmZIP3
ZmZIP4
ZmZIP5
ZmZIP6
ZmZIP7
pH5.5
ZmZIP8
ZmIRT1
OsZIP5
OsZIP8
OsIRT1
DY1455
pFL61
B
MES (mM)
50
50
50
FeCl3 (μM)
0
50
100
ZmZIP1
ZmZIP2
ZmZIP3
ZmZIP4
ZmZIP5
ZmZIP6
ZmZIP7
ZmZIP8
ZmIRT1
OsZIP5
OsZIP8
OsIRT1
DY1455
pH5.8
pFL61
