# Supplementary material for: Identification and characterization of the zinc-regulated transporters, iron-regulated transporter-like protein (ZIP) gene family in maize
Source: BMC Plant Biol. 2013 Aug 8;13:114. doi: 10.1186/1471-2229-13-114 (PMC3751942; doi:10.1186/1471-2229-13-114)
Supplement: Additional file 7 — Putative cis-elements in the 2-Kb upstream promoter region of translation start site in ZmZIP genes. [file 1471-2229-13-114-S7.docx]

| Factor | *ZmIRT1* | *ZmZIP4* | *ZmZIP5* | *ZmZIP6* |
| --- | --- | --- | --- | --- |
| -300ELEMENT  AACACOREOSGLUB1  RYREPEATBNNAPA  Skn-1_motif  DPBFCOREDCDC3DC3  -300CORE | 3  -  -  3  3  - | -  1  -  2  8  - | 2  1  2  4  3  - | 1  1  1  7  5  2 |

An *in silico* promoter analysis of *ZmZIP* genes were made with Plantcare (<http://bioinformatics.psb.ugent.be/webtools/plantcare/html/>), -300ELEMENT, AACACOREOSGLUB1, Skn-1_motif, and -300CORE are involved in controlling the endosperm-specific expression; RYREPEATBNNAPA is required for seed specific expression; DPBFCOREDCDC3DC3 is involved in controlling the embryo-specific expression.
