# Supplementary material for: Identification and characterization of the zinc-regulated transporters, iron-regulated transporter-like protein (ZIP) gene family in maize
Source: BMC Plant Biol. 2013 Aug 8;13:114. doi: 10.1186/1471-2229-13-114 (PMC3751942; doi:10.1186/1471-2229-13-114)
Supplement: Additional file 8 — Primers used in gene cloning, vector construction and real-time RT-PCR analysis. [file 1471-2229-13-114-S8.docx]

| **Primer**  **names** | **Primer sequences** | **Enzyme**  **(underlined)** |
| --- | --- | --- |
| Primers for cloning | | |
| ZmZIP1F | 5'-GCGGCCGCATGCGCCGCCAAAGCCT-3' | *NotI* |
| ZmZIP1R | 5'-GCGGCCGCTTATTCTACCAGAGAAATGCCTAGAGCG-3' | *NotI* |
| ZmZIP2F | 5'-TACGTAATGGCCCGCGCCAC-3' | *SnaBI* |
| ZmZIP2R | 5'-TACGTATCAGGTGTCCCATATCATGACG-3' | *SnaBI* |
| ZmZIP3F | 5'-CCCGGGATGGGAGCTGTGAAGCATACATTG-3' | *SmaI* |
| ZmZIP3R | 5'-GGTACCCTATGCCCATATAGCAAGCATGGAC-3' | *KpnI* |
| ZmZIP4F | 5'-ATGGACGCCACGCGAGTTCG-3' |  |
| ZmZIP4R | 5'-CTACGCCCATTTGGCGAGCAAC-3' |  |
| ZmZIP5F | 5'-GAATTCATGCCGCTCCTCGAGGAGAT-3' | *EcoRI* |
| ZmZIP5R | 5'-GCGGCCGCGAGCTCCTAAGCCCAGATGGCTAGAGATGCC-3' | *NotI,SacI* |
| ZmZIP6F | 5'-TACGTAATGTCCGGCACCGGGT-3' | *SnaBI* |
| ZmZIP6R | 5'-TACGTACTATGCCCAGAGAGCTAATACCG-3' | *SnaBI* |
| ZmZIP7F | 5'-TCTAGAATGGTTCTCGCCGGCCTC-3' | *XbaI* |
| ZmZIP7R | 5'-GAGCTCTCAAGCCCATATTGCAAGTGATGACATAG-3' | *SacI* |
| ZmZIP8F | 5'-CCCGGGATGGCCATGAGGCCACG-3' | *SmaI* |
| ZmZIP8R | 5'-GAGCTCCTAGGCCCACTTGGCCAGC-3' | *SacI* |
| ZmIRT1F | 5'-GCGGCCGCTCTAGAATGTCTTGGCGGCGAAACC-3' | *NotI,XbaI* |
| ZmIRT1R | 5'-GCGGCCGCTACGTATCACGCCCACTTGGCCATGATG-3' | *NotI,SnaBI* |
| OsZIP5F | 5'-CCCGGGGAGCCATCGGCGATGGCGA-3' | *SmaI* |
| OsZIP5R | 5'-GAGCTCGTGATGGTCACTCACTCATCACGCC-3' | *SacI* |
| OsZIP8F | 5'-GCGGCCGCATGAGGACGAACACCACC-3' | *NotI* |
| OsZIP8R | 5'-GCGGCCGC CCTCTACATTAGTCCCTGAG-3' | *NotI* |
| OsIRT1F | 5'-GCGGCCGCCCCGGGATGGCGACGCCGCGGA-3' | *NotI,SmaI* |
| OsIRT1R | 5'-GCGGCCGCCCCGGGTCACGCCCACTTGGCCATG-3' | *NotI,SmaI* |
| Primers for subcellular localization | | |
| ZmZIP1GF | 5'-GAATTCATGCGCCGCCAAAGCCT-3' | *EcoRI* |
| ZmZIP1GR | 5'-TCTAGATTCTACCAGAGAAATGCCTAGAGCG-3' | *XbaI* |
| ZmZIP2GF | 5'-GAATTCATGGCCCGCGCCACCAA-3' | *EcoRI* |
| ZmZIP2GR | 5'-TCTAGAGGTGTCCCATATCATGACGACGG-3' | *XbaI* |
| ZmZIP3GF | 5'-GAATTCATGGGAGCTGTGAAGCATAC-3' | *EcoRI* |
| ZmZIP3GR | 5'-TCTAGATGCCCATATAGCAAGCATGGACAT-3' | *XbaI* |
| ZmZIP4GF | 5'-CTCGAGATGGACGCCACGCGAGTTCG-3' | *XhoI* |
| ZmZIP4GR | 5'-TCTAGACGCCCATTTGGCGAGCAAC-3' | *XbaI* |
| ZmZIP5GF | 5'-GAATTCATGCCGCTCCTCGAGGAGAT-3' | *EcoRI* |
| ZmZIP5GR | 5'-TCTAGAAGCCCAGATGGCTAGAGATGCC-3' | *XbaI* |
| ZmZIP6GF | 5'-CCATGGCGATGTCCGGCACCGGGTG-3' | *NcoI* |
| ZmZIP6GR | 5'-TCTAGATGCCCAGAGAGCTAATACCGACAT-3' | *XbaI* |
| ZmZIP7GF | 5'-GAATTCATGGTTCTCGCCGGCCTC-3' | *EcoRI* |
| ZmZIP7GR | 5'-TCTAGAAGCCCATATTGCAAGTGATGACATAG-3' | *XbaI* |
| ZmZIP8GF | 5'-GAATTCATGGCCATGAGGCCACGC-3' | *EcoRI* |
| ZmZIP8GR | 5'-TCTAGAGGCCCACTTGGCCAGCAT-3' | *XbaI* |
| ZmIRT1GF | 5'-CTCGAGATGTCTTGGCGGCGAAACC-3' | *XhoI* |
| ZmIRT1GR | 5'-TCTAGACGCCCACTTGGCCATGATG-3' | *XbaI* |
| Primers for yeast complementation | |  |
| ZmZIP1YF | 5'-GCGGCCGCATGCGCCGCCAAAGCCT-3' | *NotI* |
| ZmZIP1YR | 5'-GCGGCCGCTTATTCTACCAGAGAAATGCCTAGAGCG-3' | *NotI* |
| ZmZIP2YF | 5'-TACGTAATGGCCCGCGCCAC-3' | *SnaBI* |
| ZmZIP2YR | 5'-TACGTATCAGGTGTCCCATATCATGACG-3' | *SnaBI* |
| ZmZIP3YF | 5'-CCCGGGATGGGAGCTGTGAAGCATACATTG-3' | *SmaI* |
| ZmZIP3YR | 5'-GGTACCCTATGCCCATATAGCAAGCATGGAC-3' | *KpnI* |
| ZmZIP4YF | 5'-TACGTAATGGACGCCACGCGAGTTCG-3' | *SnaBI* |
| ZmZIP4YR | 5'-TACGTACTACGCCCATTTGGCGAGCAAC-3' | *SnaBI* |
| ZmZIP5YF | 5'-GCGGCCGCATGCCGCTCCTCGAGGAGAT-3' | *NotI* |
| ZmZIP5YR | 5'-GCGGCCGCCTAAGCCCAGATGGCTAGAGATGCC -3' | *NotI* |
| ZmZIP6YF | 5'-TACGTAATGTCCGGCACCGGGT-3' | *SnaBI* |
| ZmZIP6YR | 5'-TACGTACTATGCCCAGAGAGCTAATACCG-3' | *SnaBI* |
| ZmZIP7YF | 5'-TACGTAATGGTTCTCGCCGGCCTC-3' | *SnaBI* |
| ZmZIP7YR | 5'-TACGTATCAAGCCCATATTGCAAGTGATGACATAG-3' | *SnaBI* |
| ZmZIP8YF | 5'-TGCCATGGCCATGAGGCCAC-3' |  |
| ZmZIP8YR | 5'-CTAGGCCCACTTGGCCAGCATG-3' |  |
| ZmIRT1YF | 5'-GCGGCCGCTCTAGAATGTCTTGGCGGCGAAACC-3' | *NotI,XbaI* |
| ZmIRT1YR | 5'-GCGGCCGCTACGTATCACGCCCACTTGGCCATGATG 3' | *NotI,SnaBI* |
| OsZIP5YF | 5'-CCCGGGGAGCCATCGGCGATGGCGA-3' | *SmaI* |
| OsZIP5YR | 5'-GAGCTCGTGATGGTCACTCACTCATCACGCC-3' | *SacI* |
| OsZIP8YF | 5'-GCGGCCGCATGAGGACGAACACCACC-3' | *NotI* |
| OsZIP8YR | 5'-GCGGCCGCCCTCTACATTAGTCCCTGAG-3' | *NotI* |
| OsIRT1YF | 5'-GCGGCCGCCCCGGGATGGCGACGCCGCGGA-3' | *NotI,SmaI* |
| OsIRT1YR | 5'-GCGGCCGCCCCGGGTCACGCCCACTTGGCCATG-3' | *NotI,SmaI* |
| Primers for real-time RT-PCR | |  |
| RTZmZIP1F | 5'-CCTCTCTGCGTTGGTTGCTCT-3' |  |
| RTZmZIP1R | 5'-TTGATGGTTGTTTTCTGGTCGT-3' |  |
| RTZmZIP2F | 5'-CCACAAATGGCACGAGGTCT-3' |  |
| RTZmZIP2R | 5'-CGAAGACGGAGTGGAAGCAAA-3' |  |
| RTZmZIP3F | 5'-GCCTCTTGTTGGTGCCCTTA-3' |  |
| RTZmZIP3R | 5'-TCAACAATGAACGCTGTAGTGCT-3' |  |
| RTZmZIP4F | 5'-CCTTCTTCTCGCTCACCGCT-3' |  |
| RTZmZIP4R | 5'-AGCCTCGGGTTGCTGAAGT-3' |  |
| RTZmZIP5F | 5'-GCACATAGGCATAGCCACGC-3' |  |
| RTZmZIP5R | 5'-ACGCCCAAAGATAGCCCGAT-3' |  |
| RTZmZIP6F | 5'-CATCGCACAGGCTGGTTTTG-3' |  |
| RTZmZIP6R | 5'-TTCCAGCCGAAAGTGAACCA-3' |  |
| RTZmZIP7F | 5'-ACTAGGTGGGTGCATTGCTCAG-3' |  |
| RTZmZIP7R | 5'-TGCCAGCAGATACCGAGTCAA-3' |  |
| RTZmZIP8F | 5'-CGTGTCATCGCTCAGGTTCTTG-3' |  |
| RTZmZIP8R | 5'-CCCTCGAACATTTGGTGGAAG-3' |  |
| RTZmIRT1F | 5'-CACCACCTTCGTCGCCAT-3' |  |
| RTZmIRT1R | 5'-TGTTGCCACCCTTCCTCC-3' |  |
| ZmActin1F | 5'-ATGTTTCCTGGGATTGCCGAT-3' |  |
| ZmActin1R | 5'-CCAGTTTCGTCATACTCTCCCTTG-3' |  |
